# Presumptive First Record of *Myotis aurascens* (Chiroptera, Vespertilionidae) from China with a Phylogenetic Analysis

**DOI:** 10.3390/ani13101629

**Published:** 2023-05-12

**Authors:** Xiufeng Yang, Xingyao Chen, Xiaodong Gao, Guolei Sun, Xue Song, Huashan Dou, Honghai Zhang

**Affiliations:** 1 School of Life Science, Qufu Normal University, Qufu 273165, China; yangxf9066@163.com (X.Y.);; 2 Hulunbuir Academy of Inland Lakes in Northern Cold & Arid Areas, Hulunbuir 021000, China; 3 Hulunbuir Forestry and Grassland Bureau, Hulunbuir 021000, China

**Keywords:** *Myotis aurascens*, taxonomic status, phylogenetic analysis, genetic distance

## Abstract

**Simple Summary:**

The taxonomic status of *Myotis aurascens* has been debated. Based on morphological and molecular data, our study showed that *M. aurascens* was closely related to *M. ikonnikovi*, *M. alcathoe*, and *M. mystacinus* and was not a synonym for *M. davidii*. Therefore, the integrated analysis demonstrated that *M. aurascens* should be considered a distinct species rather than a synonym of *M. davidii*. This study clarified the taxonomic status of *M. aurascens*, which should help enrich biodiversity and improve the species determination of bats in China.

**Abstract:**

Bat groups have a high degree of species diversity, and the taxonomic status and phylogenetic relationships among bat species have always been research hotspots. Due to the fact that morphological characteristics do not always reflect the evolutionary relationships among species, mitochondrial DNA has been widely used in the study of species relationships due to its maternal inheritance pattern. *Myotis aurascens* has been suggested as a possible synonym for *M. davidii*. However, the status of this classification has been controversial. In this study, the morphological and molecular characteristics of a *M. aurascens* captured from Inner Mongolia, China, were analyzed to determine its taxonomic status. In terms of morphological features, the body weight was 6.33 g, the head and body length were 45.10 mm, the forearm length was 35.87 mm, and the tragus length was 7.51 mm. These values all fell within the species signature data range. Nucleotide skew analysis of the protein-coding genes (PCGs) suggested that only five PCGs (*ND1*, *ND2*, *COX2*, *ATP8*, and *ND4*) showed AT-skew value within the mitogenome of *M. aurascens*. Except for *ND6*, the GC-skew values of the other PCGs were negative, reflecting the preference for C and T bases compared to G and A bases. Molecular phylogenetic analyses based on mitochondrial PCGs indicated that *M. aurascens* was a distinct species from *M. davidii* and phylogenetically closer to *M. ikonnikovi*, *M. alcathoe*, and *M. mystacinus*. Genetic distance analysis also showed that *M. aurascens* and *M. davidii* were distantly related. Therefore, the integrated analysis demonstrated that *M. aurascens* should be considered a distinct species rather than a synonym of *M. davidii*. Our study could provide a reference for enriching species diversity and research on conservation in China.

## 1. Introduction

*Myotis* is one of the most diverse genera in Mammalia and includes about 90 species worldwide. *Myotis* species are widely distributed all over the world and are found on all continents except Antarctica [1,2]. Currently, 27 species of the genus and their distributions have been documented in China [3]. Most species in this genus have been well recognized on the basis of morphological and phylogenetic analyses. Nevertheless, the taxonomic statuses of several congeners remain unresolved, for instance, *Myotis aurascens* (Steppe whiskered bat; Kuzyakin, 1935, lsid: zoobank.org:pub:1F6FA9AB-88F0-4C1B-8895-6629C23C646B). *M. aurascens* is distributed in the southeast Mediterranean and extends eastward out of the region into steppe Europe and south-west Asia [4]. In addition to the IUCN description, the occurrence of *M. aurascens* has also been found in Russia, Mongolia, Korea, and Montenegro [5,6,7], and it may be one of the most common bat species in parts of Korea [8]. Its habitat tends to be forest, scrubs (including Mediterranean-type scrubs), and crevices in rocks [4]. Although the IUCN considers the habitat of *M. aurascens* to include China, it has not yet been recorded there.

There are few studies on this species at present, probably because its taxonomic status has not been resolved. In the latest version of the Handbook [2], *M. aurascens* is considered conspecific with *M. davidii* (David’s Myotis), and not a separate species. In addition, *M. aurascens* was once known as a subspecies of the whiskered bat (*Myotis mystacinus*) [7]. This may be based on morphological data, as there are only a few phylogenetic analyses based on a small number of mitochondrial genes (*Cytb* and *COI*) without exploring evolutionary relationships with the disputed species [6]. With the development of molecular biology, molecular methods are effective for differentiating between sister species with similar appearances [9,10,11]. Mitochondria, as organelles of aerobic respiration, play an important role in the process of animal adaptation to different ecological environments [12]. Mitochondrial DNA is widely used as a molecular marker in phylogenetic and evolutionary analyses due to its simple technical procedures, conservation in various organisms, and maternal inheritance pattern [13].

In this study, one *M. aurascens* individual was collected during field surveys in Hulun Lake National Nature Reserve, Inner Mongolia, China. Although we have published the mitochondrial sequences of *M. aurascens*, we only reported the sequence information of *M. aurascens* and initially explored its evolutionary status in previous studies [14]. However, we did not investigate its relationship with *M. davidii*. Here, we systematically reassessed the taxonomic status of the published *M. aurascens* and *M. davidii* by using the complete mitochondrial genome as a molecular marker to analyze the phylogenetic relationships within the genus *Myotis*. Meanwhile, the morphological characteristics, gene arrangement, nucleic acid composition, and base preference were further compared to clarify the characteristics of the mitogenome of *M. aurascens*. Molecular phylogenetic analyses support the opinion that *M. aurascens* is a distinct species and should not be classified as *M. davidii*, and this is the first report of this species in China.

## 2. Materials and Methods

### 2.1. Sample Collection and DNA Extraction

A single individual of *M. aurascens* was collected from Hulun Lake National Nature Reserve, Inner Mongolia, China, on 1 August 2018. The sampled individual was captured using mist nets during animal investigations. The morphological indexes were measured using an electronic balance (accurate to 0.01 g) and vernier calipers (accurate to 0.01 mm). The sample was frozen in an ultra-low temperature freezer. DNA was extracted from the tissue using the DNeasy Blood & Tissue Kit (QIAGEN, Beverly, MA, USA). All sample procedures and experimental methods were approved by the Qufu Normal University Institutional Animal Care and Use Committee (Permit Number: 2022002).

### 2.2. MtDNA Sequencing, Assembly, and Annotation

The library was constructed and sequenced using an Illumina MiSeq platform (Illumina, San Diego, CA, USA) with 150 paired ends. The raw reads were filtered and quality controlled to obtain clean reads for subsequent mitochondrial genome assembly. First, A5-MISeq (v. 20150522) [15] and SPAdes (v. 3.9.0) [16] were used to assemble the clean data into contig and scaffold sequences. Then, Blastn (v. 2.2.31; https://blast.ncbi.nlm.nih.gov/Blast.cgi?PROGRAM=tblastn&PAGE_TYPE=BlastSearch&BLAST_SPEC=&LINK_LOC=blasttab&LAST_PAGE=blastn, accessed on 15 May 2022) was used to compare sequences of high sequencing depth with the Nt database, and the mitochondrial sequences of each spliced result were selected. Finally, Pilon (v. 1.18) [17] software was used to correct the results to obtain the final mitochondrial sequence. After annotating the sequence using the online software Banklt, the genome was deposited in GenBank with the accession number OK053029.

### 2.3. Characteristic Analysis of Comparative Genomes

The circular mitochondrial genome map of *M. aurascens* was drawn using the online database OGDraw (https://chlorobox.mpimp-golm.mpg.de/OGDraw.html, accessed on 28 May 2022). The relative synonymous codon usage (RSCU) was calculated using CodonW (v. 1.4.2; http://bioweb.pasteur.fr/seqanal/interfaces/codonw.html, accessed on 12 December 2022) and the base composition was estimated using the BioEdit (v. 7.2.5) software [18]. The online tRNAscan-SE (http://lowelab.ucsc.edu/tRNAscan-SE, accessed on 14 December 2022) was used to infer the secondary structures. The GC-skews and AT-skews were calculated using the following formulas: AT skew = (A − T)/(A + T) and GC skew = (G − C)/(G + C) [19].

### 2.4. Phylogenetic Analysis

Phylogenetic analysis was performed by comparing the mitogenome sequences of *M. aurascens* with those of other Vespertilionidae (including 43 *Myotis*, 1 *Hypsugo*, 5 *Murina*, 3 *Nyctalus*, 3 *Pipistrellus*, 3 *Plecotus*, and 2 *Vespertilio*), *Miniopterus fuliginosus,* and *Tadarida latouchei* as outgroups of the phylogeny. The models of evolution were evaluated using corrected Akaike information criteria (AICc) in ModelTest (v. 3.7) [20] to determine the best nucleotide substitution model [21]. Two methods, RAxML [22] and MrBayes [23], were used to construct a maximum likelihood (ML) tree based on three PCGs (*ND1*, *Cytb*, and *COX1*). The RAxML tree was evaluated with a bootstrap test with 1000 generations, and the Markov chain Monte Carlo (MCMC) analyses of the Bayes tree were run for 1,000,000 generations. The PAUP [24] software was used to construct an ML tree based on 12 PCGs. StarBeast (v. 2.6.7) software [25] was used to construct phylogenetic trees for species classification to prove our viewpoint. P-distance and the maximum composite likelihood method were used to calculate the genetic distance of nine species with 1000 bootstrap replications in MEGA7 [26].

## 3. Results

### 3.1. Habitat and Morphology

**Figure 1:**
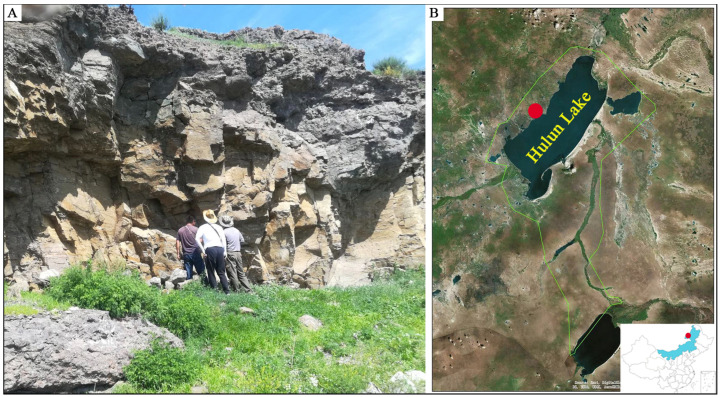
(**A**) Habitat landscape of *M. aurascens*; (**B**) sample collection site.

The sample of *M. aurascens* was collected from Hulun Lake National Nature Reserve (48°54′28.41″ N, 117°5′2.65″ E), and the habitat type was lakeshore cliffs (Figure 1). The measurement results of the external morphology showed that the body mass (BM) was 6.33 g, the forearm length (FL) was 35.87 mm, the length of the head and body (LHB) was 45.10 mm, the ear length (EL) was 13.22 mm, and the ear width (EW) was 7.25 mm (Table 1). Compared with the report of Kim [6], the tibia length (TBL), tail length (TL), and tragus length (TRL) of the Inner Mongolian specimen were shorter, at 15.11 mm, 32.70 mm, and 7.51 mm, respectively. The TL of the Chinese population was also shorter than those in Oh’s study (36.00–42.00 mm) [27]. Compared with *M. davidii*, *M. aurascens* is larger in BM, FL, LHB, EL, and EW values. Compared to *M. davidii*, the *M. aurascens* specimen was larger. PCA analysis results based on morphological data (FL, LHB, TL, and EL) supported that our study sample was *M. aurascens* (Figure S1).

### 3.2. Genome Organization

**Figure 2:**
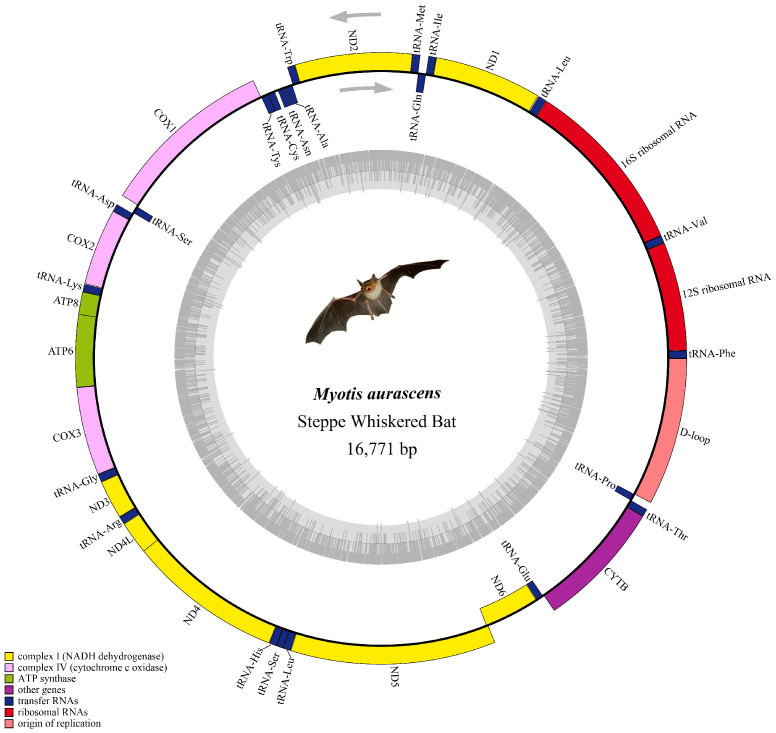
Mitochondrial genome map of *M. aurascens*.

The assembled sequence was deposited at NCBI in our previous study with the accession number OK053029 [14]. The sequence of *M. davidii* was downloaded from NCBI under accession number NC_025568.1. As shown in Figure 2, the mitogenome of *M. aurascens* was a circular DNA molecule of 16,771 bp in length, and that of *M. davidii* was 17,531 bp. Both genomes contain 37 genes, including 13 PCGs, 22 transfer RNA genes (tRNAs), two ribosomal RNA genes (rRNAs), and a D-loop. The length difference between the two species was mainly in the rRNA genes and D-loop. Most of the genes were encoded in the heavy strand (H-strand), while eight tRNAs and ND6 were encoded in the light strand (L-strand), which was similar to that of other species of the same genus [29].

The nucleotide composition of *M. aurascens* is shown in Table 2. The percentage of A + T (64.8%) was significantly higher than that of C + G (35.2%), which was considered to play a vital role in the transcription and replication of the mitochondrial genome [30]. The percentages of A + T and C + G were 63.64% and 36.33%, respectively. The analysis of the complete mitogenome nucleotide skew showed that it had a positive AT-skew value (0.046) and a negative GC-skew value (−0.262) (Table S1).

### 3.3. Characterization of Coding Genes

The total length of the 13 PCGs was 11,405 bp, which accounted for 68.0% of the whole mitogenome (Table 2). Similar to the whole genome, the average A + T content of PCGs in the mitogenome was 65.92%, ranging from 62.46% (*COX1*) to 70.41% (*ATP8*), and was higher than G + C (34.08%) in 13 PCGs. Only five PCGs had positive AT-skew values in the mitogenome of *M. aurascens* (*ND1*, *ND2*, *COX2*, *ATP8*, and *ND4*), while the GC-skew value was negative except for *ND6*, reflecting the preference for C and T bases compared to G and A bases (Table S1). All of the initiation codons of the PCGs were ATG or ATA codons, except for the *ND2* gene, which started with ATT. All of the termination codons were TAA or truncated T residues, except for the *Cytb* gene, which stopped with AGA (Table 2).

**Figure 3:**
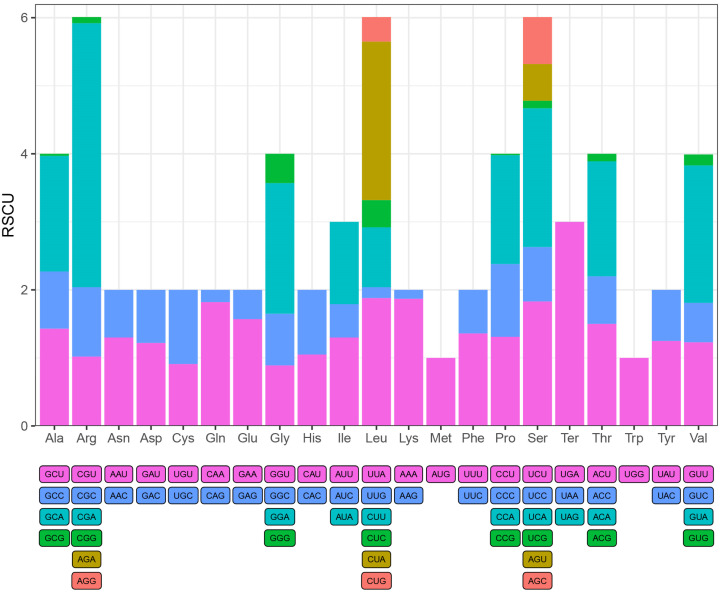
RSCU values of protein-coding genes in mitochondrial genome of *M. aurascens*.

The count and RSCU values of *M. aurascens* are shown in Table S2 and Figure 3. Among them, 26 codons were used more frequently (RSCU > 1, Table S2), and the most commonly used codons were AUU-Ile (249), CUA-Leu (236), AUA-Ile (231), UUA-Leu (190), and CUG-Phe (159). The entire length of the 22 typical tRNA genes was 1514 bp and ranged from 59 bp (Ser1) to 74 bp (Leu2). Eight of these were located on the L-strand and fourteen on the H-strand, and all of these tRNA genes were able to form the classical cloverleaf secondary structure except for seryl-tRNA (Table 2 and Figure S2).

### 3.4. Phylogenetic Analysis within Myotis

**Figure 4:**
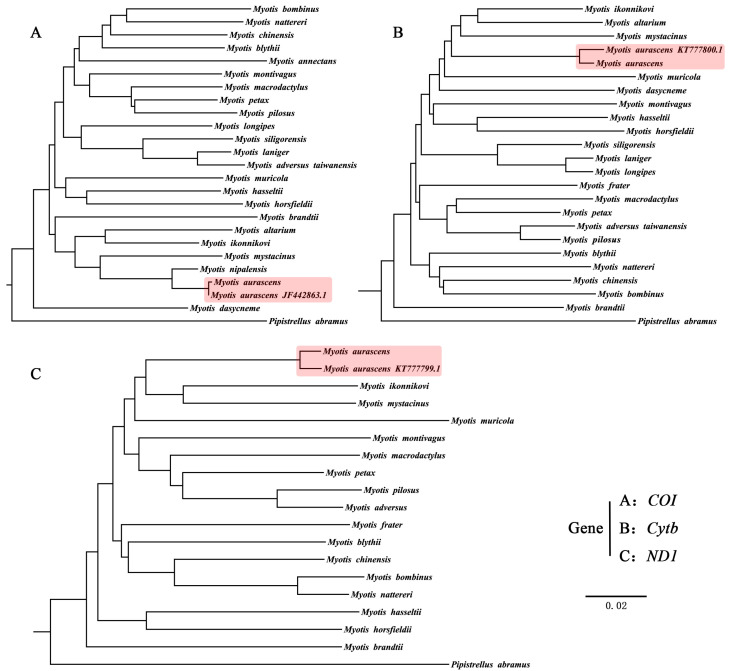
The ML analyses of phylogenetic relationships of members of the *Myotis* genus based on (**A**) *COI*, (**B**) *Cytb,* and (**C**) *ND1*.

**Figure 5:**
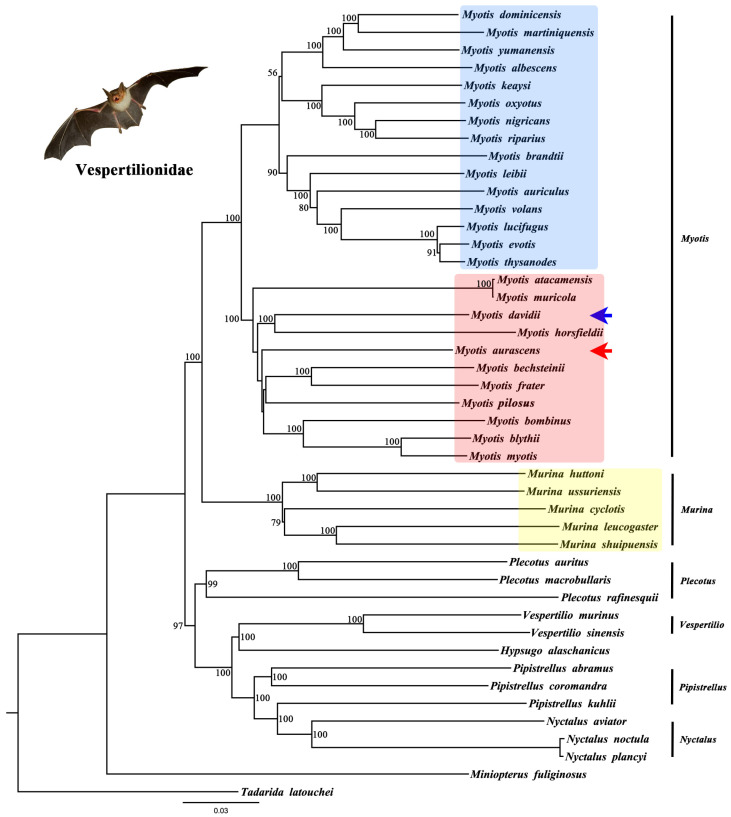
The ML analyses of phylogenetic relationships of the Vespertilionidae based on 12 PCGs. Within the Vespertilionidae, *Myotis* (blue and rose) and *Murina* (yellow) were closely related. Within the *Myotis*. *M. aurascens* (red arrow) and *M. davidii* (blue arrow) are distantly related.

Firstly, we determined if our sample was *M. aurascens* and if the sequence we downloaded was *M. davidii*. We downloaded the published sequences of the *COI*, *Cytb*, and *ND1* genes of *M. aurascens* and the *Cytb* sequence of *M. davidii* from NCBI. The phylogenetic tree results confirmed that the sequence we selected was correct (Figure 4 and Figure S3). To explore the taxonomic status of *M. aurascens* and its phylogenetic relationship with *M. davidii* and *M. mystacinus*, phylogenetic trees were constructed using all or some of the PCGs, respectively. Based on 12 PCGs (excluding *ND6*) of 45 species (Table S3), a phylogenetic tree was obtained using the ML method with 1000 replications, in which *T. latouchei* and *M. fuliginosus* were set as the outgroups (Figure 5). As a result, there were two main clades within the genus *Myotis* (blue and rose); compared with *M. davidii*, *M. aurascens* was phylogenetically closer to *M. bechsteinii*, *M. frater*, *M. pilosus*, *M. bombinus*, *M. blythii*, and *M. myotis*.

Since the mitochondrial genome of *M. mystacinus* was not available, phylogenetic trees of 64 species were constructed based on single (Table S4, Figure 4) and combined gene sequences of *ND1*, *Cytb,* and *COX1*.

**Figure 6:**
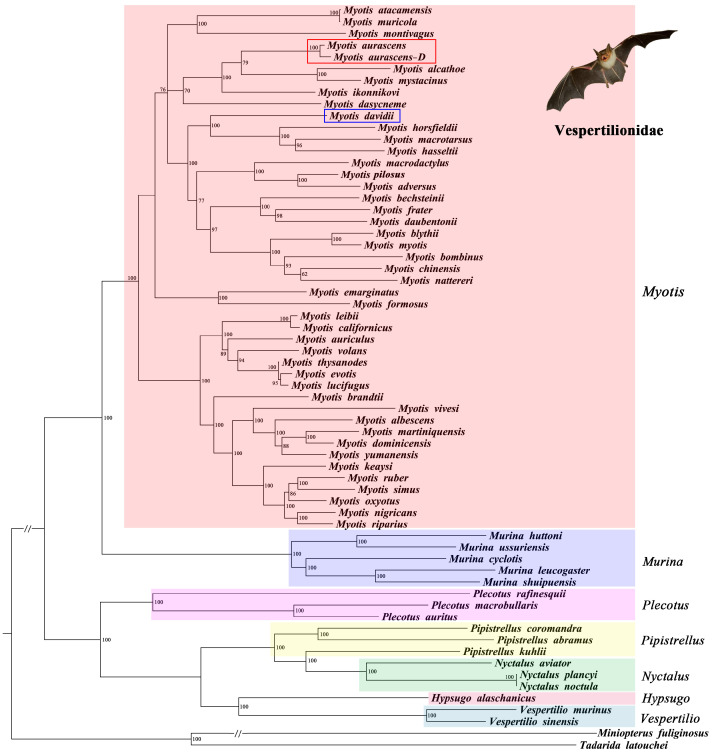
The ML analyses of phylogenetic relationships based on *ND1*, *Cytb*, and *COX1*. The number along the branch indicates the bootstrap value.

Based on *COX1* gene sequences, the phylogenetic tree showed that *M. aurascens* was closely related to *M. nipalensis*. Based on the combined sequences of the three genes, the two methods obtained different topological structures: the RAxML tree showed *M. aurascens* and *M. ikonnikovi* to be sister species and then clustered with *M. alcathoe* and *M. mystacinus* with moderate bootstrap support (Figure S4). On the contrary, the Bayes tree showed that *M. aurascens* first clustered with *M. alcathoe* and *M. mystacinus*, and then clustered with *M. ikonnikovi* with strong bootstrap support. However, the Bayes tree obtained high support values, so the result was more convincing (Figure 6). Both topological structures indicated that *M. mystacinus* and *M. davidii* were distantly related and should belong to different species. Further, StarBeast was used to construct phylogenetic trees for species classification. Several species closely related to *M. aurascens* and *M. davidii* were selected, and the results support our viewpoint (Figure S5).

### 3.5. Phylogenetic Analysis within Vespertilionidae

Further, we discussed the evolutionary relationships among the genera within Vespertilionidae. As shown in Figure 5 and Figure 6, the evolutionary tree constructed using different gene sets obtained the same topology. Within Vespertilionidae, two main clades were recovered. One clade included the genera *Myotis* and *Murina*, and the other clade included the genera *Plecotus*, *Vespertilio*, *Pipistrellus*, and *Nyctalus*. These results indicated that *Myotis* is closely related to *Murina*. *Pipistrellus* was closely related to *Nyctalus*, and *Hypsugo* was closely related to *Vespertilio*, they clustered together, then with *Plecotus,* to form the third cluster. All of these structures had strong support values.

### 3.6. Genetic Distance Analysis

To further confirm the genetic relationship between *M. aurascens* and *M. davidii*, the pairwise distances of several related species were calculated in the two phylogenetic trees. As is shown in Table 3, the pairwise distances between *M. aurascens* and *M. davidii* were 0.138 and 0.161, higher than those between any other species. The pairwise distance between *M. aurascens* and *M. pilosus* was the smallest (0.133 and 0.154), indicating that the two species are closely related.

The pairwise distances between *M. aurascens* and *M. davidii* calculated based on three genes were 0.134 and 0.156, higher than *M. ikonnikovi*, *M. alcathoe*, and *M. mystacinus* (Table 4). This research showed that *M. aurascens* and *M. davidii* were distantly related and clearly need to be classified as different species.

## 4. Discussion

At present, there is no report on the study of *M. aurascens* in China, which may be due to its unclear taxonomic status. Due to their similar appearance, many previous studies have confused it with *M. mystacinus*. However, through morphological and molecular studies, it was confirmed that *M. aurascens* and *M. mystacinus* are different species [7]. Our results also supported this view (Figure 6).

However, the current classification system classifies *M. aurascens* as *M. davidii*, not as a separate species [2]. Based on the phylogenetic tree constructed using a single mitochondrial gene (*COX1*, Figure 4A), we found that the closest genetic relationship was between *M. aurascens* and *M. nipalensis* (Nepal Myotis). In China, *M. nipalensis* is mainly distributed in Qinghai, Gansu, Xinjiang, Hubei, Jiangsu, and Tibet [31,32]. Although the geographical locations of the two species is far apart, *M. nipalensis* was previously considered a subspecies of *M. mystacinus* [33] and was listed as an independent species later [34]. Therefore, it can be inferred that there are certain similarities between the two species in morphology and taxonomic status, which explains why they have the closest genetic relationship in the phylogenetic tree.

According to the phylogenetical analysis of 12 PCGs of 45 species, *M. aurascens* showed a closer relationship with *M. bechsteinii*, *M. frater*, *M. pilosus*, *M. bombinus*, *M. blythii*, and *M. myotis* compared with *M. davidii*. Based on three PCGs (*ND1*, *Cytb,* and *COX1*) of 64 species compared with *M. davidii*, the topological structures indicated that *M. aurascens* was phylogenetically closer to *M. ikonnikovi, M. alcathoe,* and *M. mystacinus*. Both topological structures indicated that *M. mystacinus* and *M. davidii* are distantly related. Genetic distance analysis results showed that the pairwise distances between *M. aurascens* and *M. davidii* were 0.152, 0.182 (12 PCGs), and 0.134, 0.156 (3 PCGs), higher than those between any other species. The results of the above molecular studies indicate that *M. aurascens* and *M. davidii* are distantly related and clearly need to be classified as different species. Within the Vespertilionidae, *Myotis* and *Murina* are closely related, while *Plecotus*, *Vespertilio*, *Pipistrellus,* and *Nyctalus* form a single branch. In conclusion, we suggest that *M. aurascens* should be recognized as a separate species. In addition, we also briefly discussed the inter-genus relationships within the Vespertilionids, and the results showed that *Myotis* were the closest relatives to *Murina*.

However, a comparative analysis of molecular and morphological data with the type specimens of *M. aurascens* and *M. davidii* was lacking. Therefore, we could not conclude that our research subjects were *M. aurascens* and *M. davidii*. However, by combining our results with the previously published molecular data of *M. aurascens* and *M. davidii*, we have clear evidence that they are two different species. Next, we need to collect molecular and morphological data on the type specimen to prove our hypothesis.

*M. aurascens* is mainly distributed in grasslands and forests, and they roost mainly in rock crevices [34]. Using radio-tracking techniques, Korean researchers found that the main range and foraging sites of *M. aurascens* were concentrated near water bodies, while they mainly inhabit forests and nearby areas during the day [35]. In this study, the *M. aurascens* specimen was captured in crevices of the coastal cliffs of Hulun Lake (Figure 1), and this habitat type is consistent with previous descriptions. Combined with previous surveys, we found that *M. aurascens* was distributed near the Shuanmazhuang protection stations and Hulungu in Hulun Lake National Nature Reserve, Inner Mongolia, China.

Since *M. aurascens* is located in the Hulun Lake National Nature Reserve, its habitat and range are well protected. Although samples were collected several times in the vicinity, the range of *M. aurascens* is narrow and the number is small compared to the other locally distributed *Vespertilio sinensis* and *Vespertilio murinus*. This suggests that further comprehensive and in-depth surveys of the species should be carried out to discover more locations in this region. Habitat degradation due to human activities (e.g., grazing) and climate change are impacting the population of *M. aurascens*. Hence, it was urgent for us to understand its population status and suggest strategies for conservation of the species.

## 5. Conclusions

Bats not only play important ecological and economic roles in pest control and plant pollination but also have important scientific research value. Hence, it is urgent for us to understand their population status and establish a monitoring network to ensure sustainable bat populations in China. In this study, based on phenotypic characteristics, molecular phylogenetic trees, and genetic distances, we found that *M. aurascens* should be considered a distinct species rather than a synonym of *M. davidii*. The *M. aurascens* we discovered mainly lives in the cliff crevices of Hulunbuir Grassland in Inner Mongolia, China. Therefore, this region should strengthen its distribution area survey and expand research to neighboring provinces to find the true distribution of this species.

## Figures and Tables

**Table 1:** Comparisons of external measurements of *M. aurascens* and *M. davidii* from different studies.

Measurement Index (g, mm)	*M. aurascens*			*M. davidii*
	*n* = 1 (This Study)	*n* = 10 [6]	*n* = 7 [27]	*n* = 3 * [28]
Body mass (BM)	6.33	5.70–7.10	-	4.50–5.00
Forearm length (FL)	35.87	34.71–38.21	33.80–37.30	34.00–34.54
Length of head and body (LHB)	45.10	42.64–46.40	40.80–48.00	38.00–41.00
Tibia length (TBL)	15.11	16.22–18.04	15.10–17.47	15.00–15.11
Tail length (TL)	32.70	41.03–45.26	36.00–42.00	31.00–35.00
Ear length (EL)	13.22	13.06–14.98	11.00–15.00	11.00–15.00
Ear width (EW)	7.25	-	-	5.51
Tragus length (TRL)	7.51	7.64–8.88	6.30–9.00	5.09
Length of hindfoot (LHF)	7.15	6.42–7.68	7.00–10.00	7.62–9.50
Wing length (WL)	91.67	-	-	-
Wing span (WS)	216.44	-	-	-
Third metacarpal length (Mc III)	28.31	-	-	31.94
Length of first phalanx of the third digit (III1)	10.27	-	-	12.02
Length of second phalanx of the third digit III2)	15.55	-	-	9.06
Fourth metacarpal length (Mc IV)	27.80	-	-	30.08
Length of first phalanx of the fourth digit IV1)	7.85	-	-	-
Length of second phalanx of the fourth digit (IV2)	7.49	-	-	-
Fifth metacarpal length (Mc V)	29.55	-	-	32.67
Length of first phalanx of the fifth digit (V1)	16.46	-	-	8.37

* Some morphological data measure only one individual.

**Table 2:** Organization of the *M. aurascens* mitochondrial genome.

Gene	Strand	Location	Size (bp)	Base Composition (%)				Start Codon	Stop Codon
				A(U)	C	G	T		
*tRNA^Phe^*	H	1–68	68	36.76	22.06	16.18	25.00	-	-
*12S rRNA*	H	69–1034	966	36.96	21.22	17.49	24.33	-	-
*tRNA^Val^*	H	1035–1103	69	39.13	17.39	13.04	30.43	-	-
*16S rRNA*	H	1103–2670	1568	38.90	18.56	15.68	25.96	-	-
*tRNA^Leu2^*	H	2672–2746	75	30.67	18.67	21.33	29.33	-	-
*ND1*	H	2752–3707	956	32.74	23.85	11.09	32.32	ATG	TA-
*tRN^AIle^*	H	3708–3776	69	34.78	11.59	17.39	36.23	-	-
*tRNA^Gln^*	L	3774–3847	74	29.73	10.81	24.32	35.14	-	-
*tRNA^Met^*	H	3848–3916	69	28.99	24.64	18.84	27.54	-	-
*ND2*	H	3917–4958	1042	39.25	25.24	7.58	27.93	ATT	T--
*tRNA^Trp^*	H	4959–5026	68	33.82	22.06	16.18	27.94	-	-
*tRNA^Ala^*	L	5032–5100	69	28.99	11.59	21.74	37.68	-	-
*tRNA^Asn^*	L	5102–5174	73	24.66	13.70	21.92	39.73	-	-
*tRNA^Cys^*	L	5207–5272	66	27.27	21.21	24.24	27.27	-	-
*tRNA^Tyr^*	L	5273–5341	69	36.23	17.39	20.29	26.09	-	-
*COX1*	H	5343–6887	1545	27.06	20.65	16.89	35.40	ATG	TAA
*tRNA^Ser2^*	L	6901–6969	69	24.64	15.94	24.64	34.78	-	-
*tRNA^Asp^*	H	6977–7043	67	37.31	11.94	13.43	37.31	-	-
*COX2*	H	7044–7727	684	33.92	21.93	12.57	31.58	ATG	TAA
*tRNA^Lys^*	H	7731–7798	68	35.29	19.12	14.71	30.88	-	-
*ATP8*	H	7799–8002	204	40.22	22.55	6.86	30.39	ATG	TAA
*ATP6*	H	7960–8640	681	32.60	23.05	11.60	32.75	ATG	TAA
*COX3*	H	8640–9423	784	28.57	22.70	14.16	34.57	ATG	T--
*tRNA^Gly^*	H	9424–9493	70	37.14	17.14	14.29	31.43	-	-
*ND3*	H	9494–9840	347	30.84	20.17	11.24	37.75	ATA	TA-
*tRNA^Arg^*	H	9841–9909	69	42.03	8.70	8.70	40.58	-	-
*ND4L*	H	9911–10,207	297	28.28	22.56	12.12	37.04	ATG	TAA
*ND4*	H	10,201–11,579	1379	33.14	23.42	10.51	32.92	ATG	TA-
*tRNA^His^*	H	11,580–11,647	68	45.59	8.82	10.29	35.29	-	-
*tRNA^Ser1^*	H	11,647–11,705	59	33.90	20.34	16.95	28.81	-	-
*tRNA^Leu1^*	H	11,706–11,775	70	38.57	14.29	20.00	27.14	-	-
*ND5*	H	11,777–13,597	1821	33.44	22.73	10.16	33.66	ATA	TAA
*ND6*	L	13,581–14,105	525	24.19	6.67	25.71	43.43	ATA	TAA
*tRNA^Glu^*	L	14,108–14,176	69	28.99	14.49	21.74	34.78	-	-
*Cytb*	H	14,184–15,323	1140	29.82	23.95	13.07	33.16	ATG	AGA
*tRNA^Thr^*	H	15,324–15,393	70	37.14	14.29	18.57	30.00	-	-
*tRNA^Pro^*	L	15,393–15,458	66	24.24	10.61	27.27	37.88	-	-
*D-loop*	H	15,459–16,771	1313	34.96	23.76	14.09	27.19	-	-
Total		16,771		33.93	22.17	12.96	30.94		

**Table 3:** ML distances (above the diagonal) and P-distances (below the diagonal) for 12 PCG sequences of *M. aurascens*.

Species	*M.* *aurascens*	*M. brandtii*	*M. davidii*	*M. muricola*	*M. bombinus*	*M. frater*	*M.* *horsfieldii*	*M.* *myotis*	*M.* *pilosus*
*M. aurascens*	-	0.182	0.161	0.177	0.168	0.163	0.187	0.158	0.154
*M. brandtii*	0.152	-	0.195	0.213	0.200	0.199	0.214	0.193	0.19
*M. davidii*	0.138	0.161	-	0.188	0.178	0.168	0.176	0.163	0.163
*M. muricola*	0.149	0.172	0.156	-	0.192	0.186	0.205	0.186	0.181
*M. bombinus*	0.143	0.163	0.150	0.159	-	0.172	0.201	0.135	0.168
*M. frater*	0.140	0.164	0.143	0.155	0.146	-	0.198	0.163	0.164
*M. horsfieldii*	0.156	0.172	0.148	0.167	0.165	0.163	-	0.195	0.188
*M. myotis*	0.136	0.159	0.139	0.155	0.119	0.140	0.161	-	0.162
*M. pilosus*	0.133	0.157	0.140	0.151	0.143	0.140	0.156	0.139	-

**Table 4:** ML distances (above the diagonal) and P-distances (below the diagonal) for *ND1*, *Cytb*, and *COX1* gene sequences of *M. aurascens*.

Species	*M.* *aurascens*	*M. ikonnikovi*	*M. alcathoe*	*M. mystacinus*	*M. davidii*	*M. dasycneme*	*M. montivagus*	*M. muricola*	*M. daubentonii*
*M. aurascens*	-	0.126	0.148	0.129	0.156	0.154	0.174	0.169	0.168
*M. ikonnikovi*	0.112	-	0.16	0.138	0.165	0.151	0.17	0.167	0.172
*M. alcathoe*	0.128	0.137	-	0.088	0.166	0.174	0.179	0.183	0.172
*M. mystacinus*	0.114	0.120	0.080	-	0.176	0.176	0.189	0.18	0.172
*M. davidii*	0.134	0.140	0.140	0.147	-	0.168	0.17	0.184	0.171
*M. dasycneme*	0.132	0.129	0.147	0.147	0.142	-	0.174	0.174	0.175
*M. montivagus*	0.146	0.143	0.150	0.157	0.143	0.145	-	0.184	0.18
*M. muricola*	0.143	0.140	0.151	0.150	0.153	0.146	0.153	-	0.188
*M. daubentonii*	0.142	0.144	0.144	0.144	0.144	0.147	0.149	0.155	-

## Data Availability

Tables S3 and S4 provide the NCBI accession numbers of the species used in this study.

## Supplementary Materials

The following supporting information can be downloaded at: https://www.mdpi.com/article/10.3390/ani13101629/s1, Figure S1: PCA analysis between *M. aurascens* and *M. davidii* based on morphological data; Figure S2: The secondary structure of tRNA gene; Figure S3: The phylogenetic tree constructed from *M. davidii* data downloaded from NCBI; Figure S4: The ML analyses of phylogenetic relationship based on *ND1*, *Cytb*, and *COX1* used two methods; Figure S5: The phylogenetic trees for species classification using StarBeast; Table S1: Base composition of the mitogenomes of *M. aurascens*; Table S2: Frequency and RSCU values of codon in PCGs in the mitogenome of *M. aurascens*; Table S3: Complete mitochondrial sequence used in molecular phylogenetic analyses in this study; Table S4: *COI*, *ND1* and *Cytb* sequence used in molecular phylogenetic analyses in this study.
