# Supplementary material for: Presumptive First Record of Myotis aurascens (Chiroptera, Vespertilionidae) from China with a Phylogenetic Analysis
Source: Animals (Basel). 2023 May 12;13(10):1629. doi: 10.3390/ani13101629 (PMC10215177; doi:10.3390/ani13101629)
Supplement: Supplementary file 1 [file animals-13-01629-s001.zip › Table S1.pdf]

Table S1 Base composition of the mitogenomes of *M. aurascens*

**Gene**	A+T（%）	AT skew	GC skew
12S rRNA	61.29	0.206	-0.096
16S rRNA	64.86	0.200	-0.084
ND1	65.06	0.006	-0.365
ND2	67.18	0.169	-0.538
COX1	62.46	-0.134	-0.100
COX2	65.5	0.036	-0.271
ATP8	70.61	0.139	-0.533
ATP6	65.35	-0.002	-0.330
COX3	63.14	-0.095	-0.232
ND3	68.59	-0.101	-0.284
ND4L	65.32	-0.134	-0.301
ND4	66.06	0.003	-0.380
ND5	67.1	-0.003	-0.382
ND6	67.62	-0.285	0.588
Cytb	62.98	-0.053	-0.294
D-loop	62.15	0.125	-0.255
Total	64.87	0.046	-0.262
