# Supplementary material for: Presumptive First Record of Myotis aurascens (Chiroptera, Vespertilionidae) from China with a Phylogenetic Analysis
Source: Animals (Basel). 2023 May 12;13(10):1629. doi: 10.3390/ani13101629 (PMC10215177; doi:10.3390/ani13101629)
Supplement: Supplementary file 1 [file animals-13-01629-s001.zip › Table S2.pdf]

Table S2 Frequency and RSCU values of codon in PCGs in the mitogenome of *M. aurascens*.

AA	Codon	Count	RSCU	AA	Codon	Count	RSCU
Ala	GCU	85	1.43	Leu	UUA	190	1.88
	GCC	50	0.84		UUG	16	0.16
	GCA	101	1.7		CUU	89	0.88
	GCG	2	0.03		CUC	41	0.4
Arg	CGU	11	1.02		CUA	236	2.33
	CGC	11	1.02		CUG	36	0.36
	CGA	42	3.88	Phe	UUU	159	1.36
	CGG	1	0.09		UUC	74	0.64
Asn	AAU	99	1.3	Pro	CCU	64	1.31
	AAC	53	0.7		CCC	52	1.07
Asp	GAU	41	1.22		CCA	78	1.6
	GAC	26	0.78		CCG	1	0.02
Cys	UGU	10	0.91	Ser	UCU	85	1.83
	UGC	12	1.09		UCC	37	0.8
Gln	CAA	81	1.82		UCA	95	2.04
	CAG	8	0.18		UCG	5	0.11
	GAA	76	1.57		AGU	25	0.54
	GAG	21	0.43		AGC	32	0.69
Gly	GGU	48	0.89	Ter	UGA	97	3
	GGC	41	0.76	Thr	ACU	120	1.5
	GGA	103	1.92		ACC	56	0.7
	GGG	23	0.43		ACA	135	1.69
His	CAU	50	1.05		ACG	9	0.11
	CAC	45	0.95	Trp	UGG	7	1
Ile	AUU	249	1.3	Tyr	UAU	88	1.25
	AUC	94	0.49		UAC	53	0.75
	AUA	231	1.21	Val	GUU	53	1.23
Lys	AAA	89	1.87		GUC	25	0.58
	AAG	6	0.13		GUA	87	2.02
Met	AUG	30	1		GUG	7	0.16
