# Supplementary material for: Presumptive First Record of Myotis aurascens (Chiroptera, Vespertilionidae) from China with a Phylogenetic Analysis
Source: Animals (Basel). 2023 May 12;13(10):1629. doi: 10.3390/ani13101629 (PMC10215177; doi:10.3390/ani13101629)
Supplement: Supplementary file 1 [file animals-13-01629-s001.zip › Table S3.pdf]

Table S3 Complete mitochondrial sequence used in molecular phylogenetic analyses in this study

Accession Number	Scientific Name	Accession Number	Scientific Name
NC_036312.1	*Myotis dominicensis*	KU521385.1	*Murina huttoni*
NC_036328.1	*Myotis martiniquensis*	NC_021119.1	*Murina ussuriensis*
NC_036319.1	*Myotis yumanensis*	MK747248.1	*Murina cyclotis*
NC_036327.1	*Myotis albescens*	NC_025949.1	*Murina leucogaster*
NC_036314.1	*Myotis keaysi*	MK747249.1	*Murina shuipuensis*
NC_036316.1	*Myotis oxyotus*	HM164052.1	*Plecotus auritu*
NC_036318.1	*Myotis nigricans*	NC_027977.1	*Plecotus macrobullaris*
NC_036317.1	*Myotis riparius*	NC_016872.1	*Plecotus rafinesquii*
NC_025308.1	*Myotis brandtii*	NC_033347.1	*Vespertilio murinus*
NC_036321.1	*Myotis leibii*	KM092493.1	*Vespertilio sinensis*
NC_036320.1	*Myotis auriculus*	MK135784.1	*Hypsugo alaschanicus*
NC_036326.1	*Myotis volans*	KX355640.1	*Pipistrellus abramus*
MF143491.1	*Myotis lucifugus*	NC_029191.1	*Pipistrellus coromandra*
NC_036313.1	*Myotis evotis*	KU058655.1	*Pipistrellus kuhlii*
NC_036323.1	*Myotis thysanodes*	MK167360.1	*Nyctalus aviator*

NC_036324.1	*Myotis atacamensis*	NC_027237.1	*Nyctalus noctula*
NC_029422.1	*Myotis muricola*	NC_041160.1	*Nyctalus plancyi*
NC_025568.1	*Myotis davidii*	NC_044489.1	*Miniopterus fuliginosus*
NC_036325.1	*Myotis horsfieldii*	MK177282.1	*Tadarida latouchei*
NC_034227.1	*Myotis bechsteinii*		
NC_041638.1	*Myotis frater*		
NC_056111.1	*Myotis ricketti*		
NC_029342.1	*Myotis bombinus*		
MT588108.1	*Myotis blythii*		
KT901455.1	*Myotis myotis*		
