# Supplementary material for: Presumptive First Record of Myotis aurascens (Chiroptera, Vespertilionidae) from China with a Phylogenetic Analysis
Source: Animals (Basel). 2023 May 12;13(10):1629. doi: 10.3390/ani13101629 (PMC10215177; doi:10.3390/ani13101629)
Supplement: Supplementary file 1 [file animals-13-01629-s001.zip › Table S4.pdf]

Table S4 *COI*, *ND1* and *Cytb* sequence used in molecular phylogenetic analyses in this study

COI		*ND1*		*Cytb*	
Accession Number	Scientific Name	Accession Number	Scientific Name	Accession Number	Scientific Name
HQ580336.1	*Myotis bombinus*	KT777799.1	*Myotis aurascens*	AB106603.1	*Myotis ikonnikovi*
JF443018.1	*Myotis nattereri*	AB106581.1	*Myotis ikonnikovi*	MF630871.1	*Myotis altarium*
HM541078.1	*Myotis chinensis*	MT628544.1*	*Myotis mystacinus*	MT628544.1*	*Myotis mystacinus*
MT407289.1	*Myotis blythii*	AY699871.1	*Myotis muricola*	KT777800.1	*Myotis aurascens*
HM541011.1	*Myotis annectans*	AY033972.1	*Myotis montivagus*	AY665144.1	*Myotis muricola*
HM541125.1	*Myotis montivagus*	AB106582.1	*Myotis macrodactylus*	AF376846.1	*Myotis dasycneme*
HQ580338.1	*Myotis macrodactylus*	KT199099.1	*Myotis petax*	AF376858.1	*Myotis montivagus*
HQ580195.1	*Myotis petax*	GU372853.1	*Myotis pilosus*	AF376850.1	*Myotis hasseltii*
HM541134.1	*Myotis pilosus*	GU372854.1	*Myotis adversus*	KP187907.1	*Myotis horsfieldii*
MN339186.1	*Myotis longipes*	AB106571.1	*Myotis frater*	FJ215679.1	*Myotis siligorensis*
JF443978.1	*Myotis siligorensis*	AF401457.1	*Myotis blythii*	MF630874.1	*Myotis laniger*
JN312059.1	*Myotis laniger*	AB106567.1	*Myotis chinensis*	MH183152.1	*Myotis longipes*
HM541172.1	*Myotis adversus taiwanensis*	NC_029342.1	*Myotis bombinus*	KX467612.1	*Myotis frater*
GU684805.1	*Myotis muricola*	AB106584.1	*Myotis nattereri*	AB085736.1	*Myotis macrodactylus*

HM541097.1	*Myotis hasseltii*	AY033973.1	*Myotis hasseltii*	EF555237.1	*Myotis petax*
JF443984.1	*Myotis horsfieldii*	AY033970.1	*Myotis horsfieldii*	KF312497.1	*Myotis adversus taiwanensis*
JF442928.1	*Myotis brandtii*	HQ529624.1	*Myotis brandtii*	MG570071.1	*Myotis ricketti*
JF443976.1	*Myotis altarium*	AB079821.1	*Pipistrellus abramus*	KX467606.1	*Myotis blythii*
HQ974652.1	*Myotis ikonnikovi*			MT992630.1	*Myotis nattereri*
MT628544.1*	*Myotis mystacinus*			AB106588.1	*Myotis chinensis*
JX008074.1	*Myotis nipalensis*			KX467609.1	*Myotis bombinus*
JF442863.1	*Myotis aurascens*			AY665168.1	*Myotis brandtii*
JF442936.1	*Myotis dasycneme*			AB085739.2	*Pipistrellus abramus*
JF444018.1	*Pipistrellus abramus*				
