# Supplementary figures and images for: Presumptive First Record of Myotis aurascens (Chiroptera, Vespertilionidae) from China with a Phylogenetic Analysis
Source: Animals (Basel). 2023 May 12;13(10):1629. doi: 10.3390/ani13101629 (PMC10215177; doi:10.3390/ani13101629)

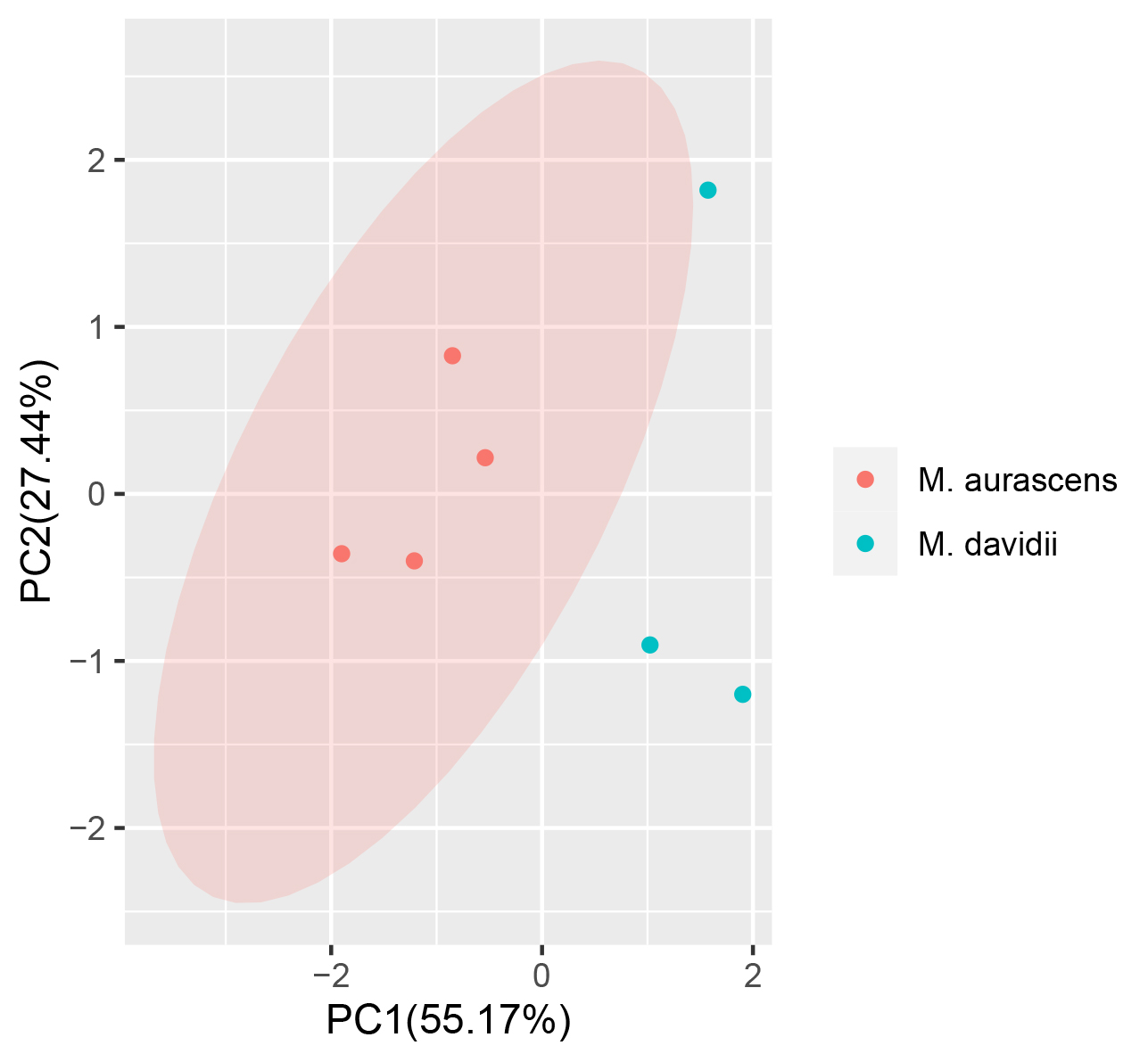

Supplement: Supplementary file 1 [file animals-13-01629-s001.zip › Figure S1. PCA analysis between M. aurascens and M. davidii based on morphological data.jpg]

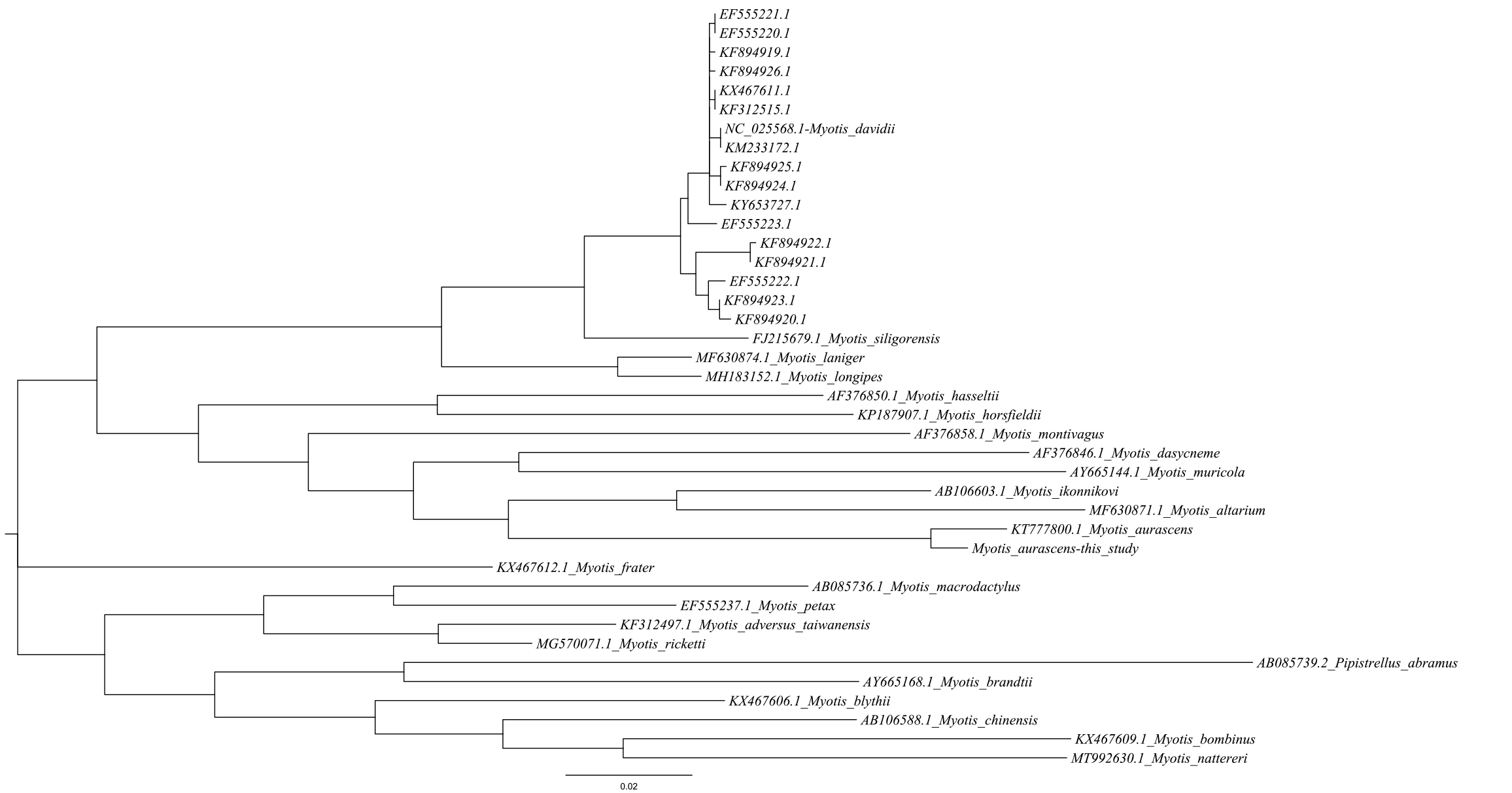

Supplement: Supplementary file 1 [file animals-13-01629-s001.zip › Figure S3. The phylogenetic tree constructed from M. davidii data downloaded from NCBI.jpg]

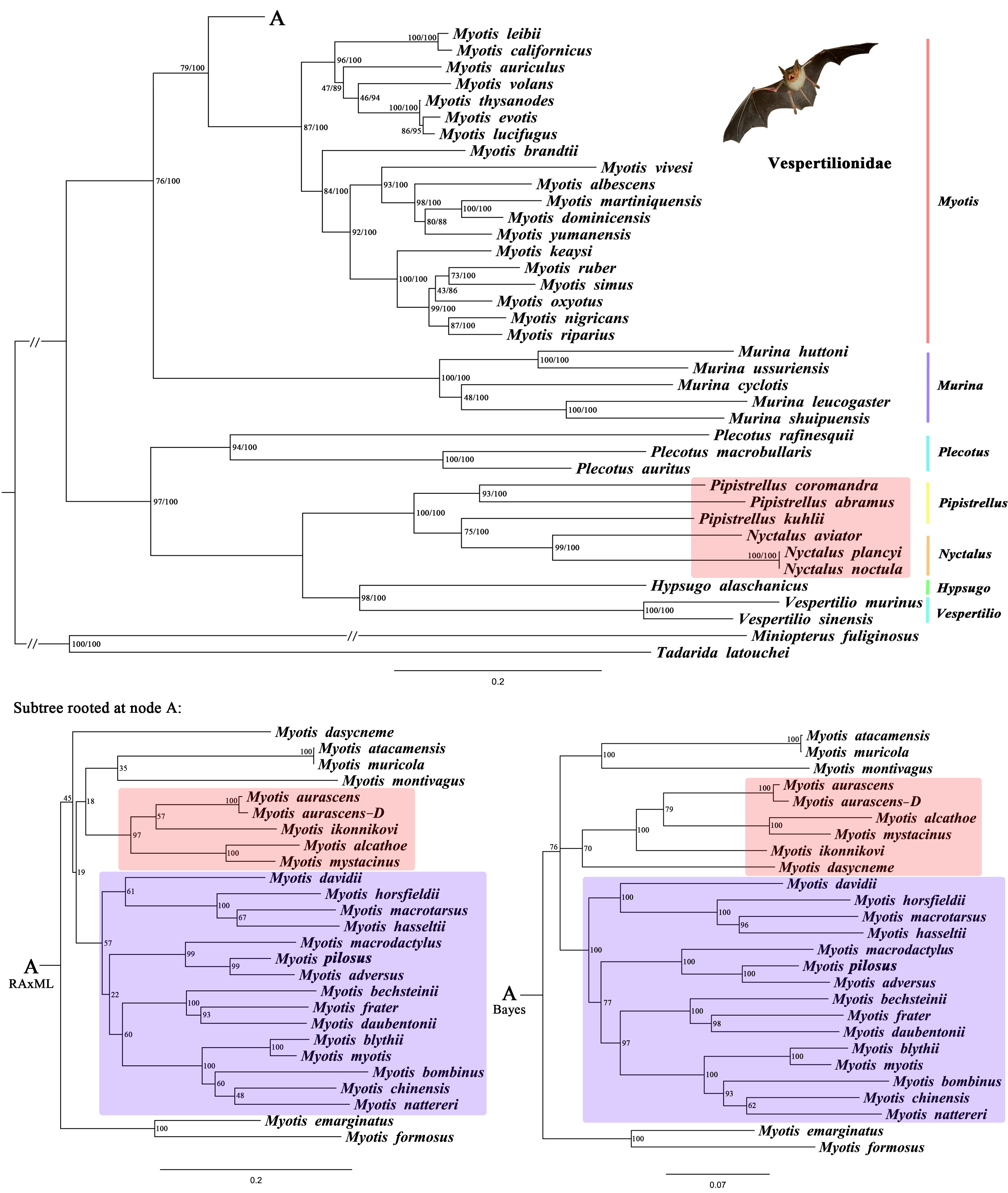

Supplement: Supplementary file 1 [file animals-13-01629-s001.zip › Figure S4. The ML analyses of phylogenetic relationship based on ND1, Cytb, and COX1 used two methods..jpg]

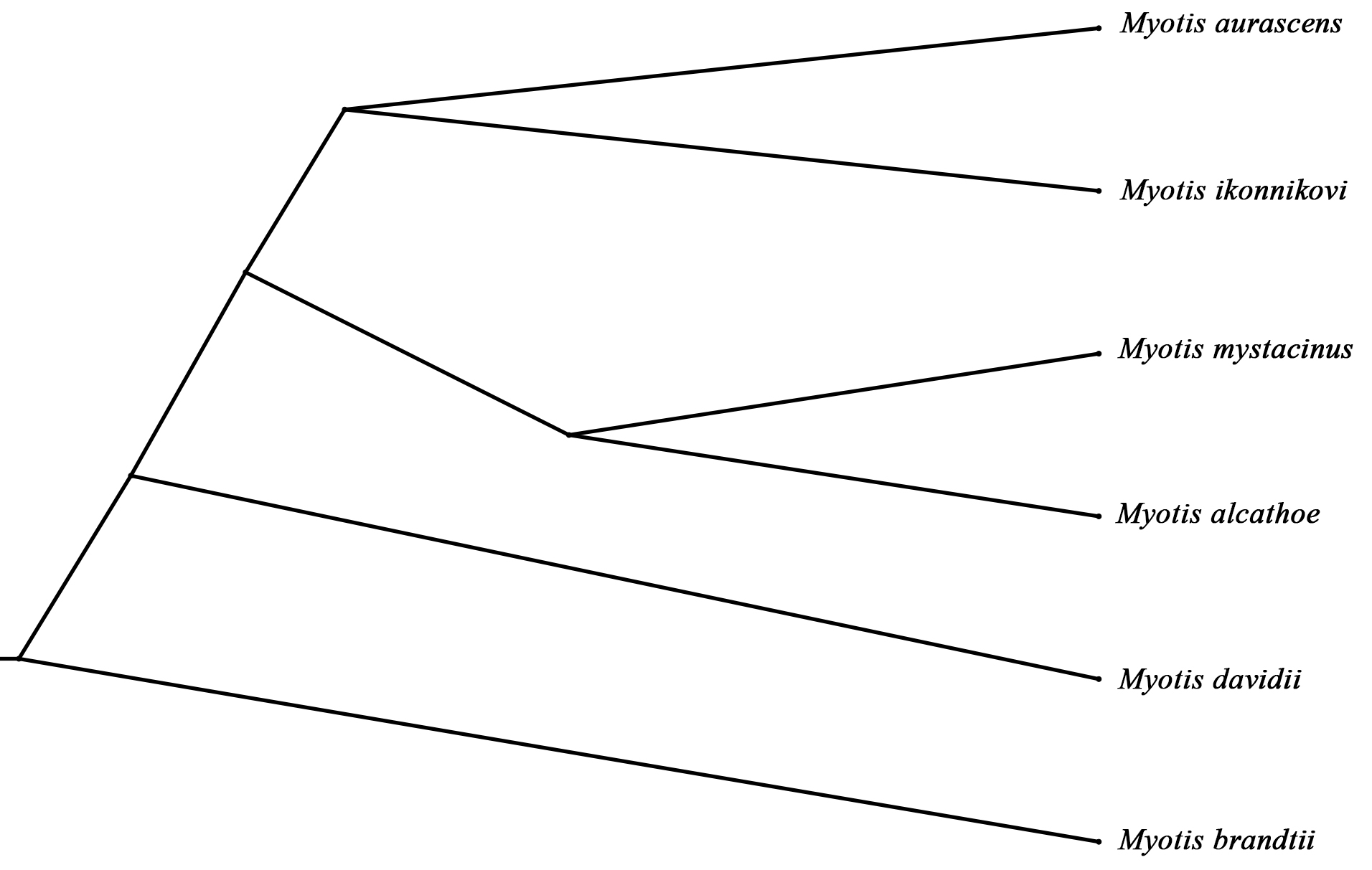

Supplement: Supplementary file 1 [file animals-13-01629-s001.zip › Figure S5. The phylogenetic trees for species classification using StarBeast..jpg]
